# Third generation cephalosporin-resistant *Klebsiella pneumoniae* thriving in patients and in wastewater: what do they have in common?

**DOI:** 10.1186/s12864-021-08279-6

**Published:** 2022-01-22

**Authors:** Jaqueline Rocha, Catarina Ferreira, Dalila Mil-Homens, Antonio Busquets, Arsénio M. Fialho, Isabel Henriques, Margarita Gomila, Célia M. Manaia

**Affiliations:** 1 Universidade Católica Portuguesa, CBQF - Centro de Biotecnologia e Química Fina – Laboratório Associado, Escola Superior de Biotecnologia, Rua Diogo Botelho 1327, 4169-005 Porto, Portugal; 2 iBB-Institute of Bioengineering and Biosciences and i4HB-Institute for Health and Bioeconomy, Instituto Superior Técnico, Lisbon, Portugal; 3 Department of Bioengineering, Instituto Superior Técnico, University of Lisbon, Lisbon, Portugal; 4 Microbiologia, Departament de Biologia, Universitat de les Illes Balears, Palma de Mallorca, Spain; 5 University of Coimbra, Department of Life Sciences, Faculty of Science and Technology, Coimbra, Portugal; 6 CESAM, University of Aveiro, Aveiro, Portugal

**Keywords:** Wastewater, Clinical, *Klebsiella pneumoniae*, Antibiotic resistance, Comparative genomics

## Abstract

**Background:**

*Klebsiella pneumoniae* are ubiquitous bacteria and recognized multidrug-resistant opportunistic pathogens that can be released into the environment, mainly through sewage, where they can survive even after wastewater treatment. A major question is if once released into wastewater, the selection of lineages missing clinically-relevant traits may occur. Wastewater (*n* = 25) and clinical (*n* = 34) 3^rd^ generation cephalosporin-resistant *K. pneumoniae* isolates were compared based on phenotypic, genotypic and genomic analyses.

**Results:**

Clinical and wastewater isolates were indistinguishable based on phenotypic and genotypic characterization. The analysis of whole genome sequences of 22 isolates showed that antibiotic and metal resistance or virulence genes, were associated with mobile genetic elements, mostly transposons, insertion sequences or integrative and conjugative elements. These features were variable among isolates, according to the respective genetic lineage rather than the origin.

**Conclusions:**

It is suggested that once acquired, clinically relevant features of *K. pneumoniae* may be preserved in wastewater, even after treatment. This evidence highlights the high capacity of *K. pneumoniae* for spreading through wastewater, enhancing the risks of transmission back to humans.

**Supplementary Information:**

The online version contains supplementary material available at 10.1186/s12864-021-08279-6.

## Background

The species *Klebsiella pneumoniae,* within the family *Enterobacteriaceae,* includes opportunistic pathogens, with ubiquitous distribution [1, 2]. The ubiquity and clinical relevance of *K. pneumoniae* is due, in part, to the genome plasticity, in which genes acquisition, such as those encoding antibiotic resistance, is a major driver [1, 3, 4]. Indeed, genes acquired by horizontal gene transfer, encoding resistance against aminoglycosides, 3^rd^ generation cephalosporins, carbapenems and fluoroquinolones [5, 6] or metals such as arsenic, copper, tellurium and mercury are frequent in *K. pneumoniae* [7]. The ubiquity and clinical relevance of *K. pneumoniae* is also due to a wide array of genes that encode functions related with adhesion, protection (capsules) or siderophore production [3]. The combination of these features and ubiquitous distribution make *K. pneumoniae* an important opportunistic pathogen, responsible for one third of the hospital infections caused by Gram-negative bacteria [6].

The ubiquity of *K. pneumoniae* is illustrated by its occurrence in healthy humans and animals, and in plants, soil, water and wastewater [1, 8, 9], suggesting that it may circulate among distinct compartments. In urban areas, domestic sewage represents the major human emission of pathogens and antibiotic resistant bacteria. Although wastewater treatment has a pivotal role for the removal of such microorganisms from sewage, an important fraction can survive, being discharged into the environment [10–12].

The occurrence of *K. pneumoniae* in treated wastewater is mainly explained by human emissions and by the capacity of members of this species to endure treatment processes. In fact, the survival or even proliferation of virulent and multidrug resistant *K. pneumoniae* discharged through sewage in the environment has been reported [13, 14]. These facts explain why identical *K. pneumoniae* sequence types (e.g. ST11, ST15, ST17, ST258 or ST147) have been reported in both clinical settings and wastewater [1, 5].

Pathogens causing infection or thriving in the environment are supposedly exposed to distinct challenges, which may hypothetically be associated with the retention or loss of specific traits and/or responsible for distinctive selective processes. A major question is if *K. pneumoniae* found in the environment retain the features observed in clinical isolates, or if some properties, like antibiotic resistance or virulence, can be lost. In accordance, this work aimed to investigate if phenotypic and genotypic traits and genome characteristics are shared by clinical and wastewater isolates. It was also aimed to compare both groups in terms of genome features associated with horizontal gene transfer. Our hypothesis was addressed using a set of isolates resistant to 3^rd^ generation cephalosporins, as this is an increasingly frequent phenotype in clinical and environmental isolates [6, 15]. A group of 59 isolates (25 from wastewater, 34 clinical) was characterized phenotypically and genotypically targeting clinically-relevant traits and a subset of these isolates (7 from wastewater, 15 clinical – 11 from patients and 4 from clinical environment) was further compared based on genome analyses. The results suggested that phylogeny, more than strains origin, may explain the profile of acquired traits.

## Results

### Preliminary characterization based on phenotype and selected genetic traits

**Fig. 1:**
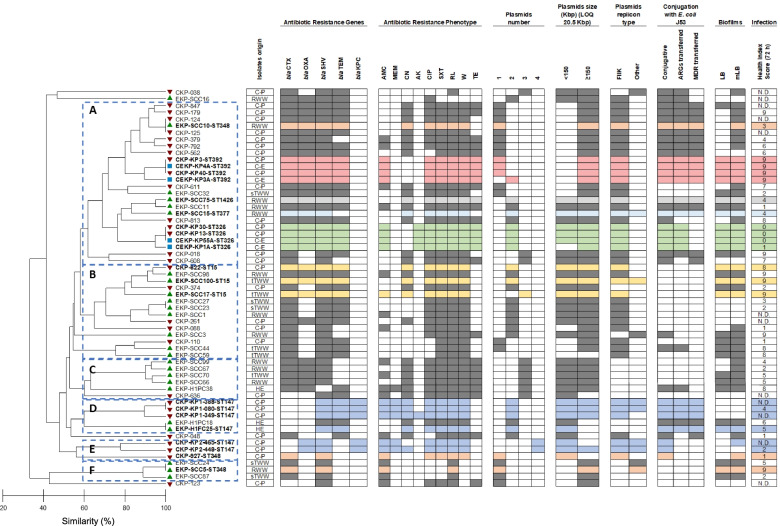
Clustering analysis using Jaccard similarity based on distinctive traits of the 3^rd^ generation cephalosporin-resistant *K. pneumoniae* isolates. Antibiotic classes tested: β-lactams and carbapenems (AMC-Amoxicillin+Clavulanate (20/10 μg); MEM-Meropenem (10 μg)); aminoglycosides (CN-Gentamicin (10 μg)); AK-Amikacin (30 μg)); quinolones (CIP-Ciprofloxacin (5 μg)); sulfonamides (SXT-Sulfamethoxazole/Trimethoprim (1.25/23.75 μg); RL-Sulfamethoxazole (25 μg); W–Trimethoprim (5 μg)); and tetracyclines (TE-Tetracycline(30 μg)). ARGs *bla*_IMP_, *bla*_VIM_ and *mcr* were also screened and were not detected in none of the isolates. All the isolates showed a resistance phenotype against amoxicillin and aztreonam. Red, green and blue symbols in the dendrogram refer to isolates obtained from patients, wastewater and clinical settings, respectively. Colors in the table refer to the trait presence and different colors refer to different sequence types. A to F letters in the dendrogram refer to the main clusters identified. C-P – clinical isolate obtained from patients; C-E – isolate obtained from clinical environment; HE – hospital effluent; RWW – raw influent wastewater; sTWW – secondary treatment effluent wastewater; tTWW – tertiary treatment effluent wastewater; N.D. - not determined; LB - Luria-Bertani; mLB - modified Luria-Bertani; ST – sequence type; CKP – clinical *K. pneumoniae*; EKP – environmental *K. pneumoniae;* CEKP – clinical environment *K. pneumoniae*

The characterization of the isolates (Table S1) based on phenotypic and genetic features evidenced identical profiles shared by clinical and wastewater isolates (Fig. 1). In general, the prevalence values observed for each of the tested characteristics were not significantly different. An exception was the trans-species conjugation (with *E. coli* J53). A total of 73% of the clinical isolates (22/30, the clinical environment was not included in this analysis) was capable of conjugating with *E. coli*, while only 40% (10/25) of wastewater had such a capacity (*p*-value = 0.016; Fisher’s exact test) (Fig. 1). Transconjugant *E. coli* J53 with acquired antibiotic resistance genes (ARGs) and multidrug resistant (MDR) phenotypes (i.e. resistance to antibiotics belonging to 3 or more classes) were more frequent for clinical than for wastewater donors (ARGs 53% - 16/30 vs. 36% -9/30; MDR 50% - 15/30 vs. 24% - 6/25, respectively), although not statistically significantly different (ARGS – *p*-value = 0.278; MDR – p-value = 0.057; Fisher’s exact test) (Table S2). The capacity to form biofilm in LB medium was significantly more frequent in wastewater (72%) than in clinical (30%) isolates (p-value = 0.003; Fisher’s exact test). In diluted LB, with half the nutrients concentration and 10 times less sodium chloride (mLB), more isolates tested positive for biofilm formation (wastewater, 84% vs. clinical, 70%) and significant differences were not observed (*p*-value = 0.341; Fisher’s exact test) (Fig. 1). The addition of cefotaxime (2 mg/L) to culture media did not affect the biofilm forming capacity. Genotypic features presented, in general, identical prevalence values in both groups. Genes encoding resistance against carbapenems and other β-lactams (*bla*_CTX_, *bla*_OXA_, *bla*_SHV_, *bla*_TEM_ and *bla*_KPC_) presented identical prevalence in clinical and in wastewater isolates (0.056 < *p*-value< 1.00; Fisher’s exact test). Also, the PFGE analysis revealed that irrespective of the origin most isolates harbored 1–3 plasmids. Plasmids larger than 150 Kbp were present in 87% of clinical and 88% of wastewater isolates, while smaller plasmids (< 150 Kbp) were more frequent in wastewater isolates (72% vs. 53% in clinical), although not statistically significant (> 150 Kbp – *p*-value = 1.00; < 150 Kbp – p-value = 0.177; Fisher’s Exact test) (Fig. 1). The *G. mellonella* Health Index scores varied between 0 and 9 (strong to no-infection capacity), being 0 and 1 more frequent in clinical (30%) than in wastewater isolates (4%) (*p*-value = 0.038; Fisher’s exact test). While the score 9 ranged 20 to 22% in both groups, scores 2–8 were more frequent in wastewater (74%) than in clinical isolates (50%), though not statistically significant (p-value = 0.127; Fisher’s exact test) (Fig. 1).

The pheno- and genotypic characterization was integrated based on a numerical taxonomy approach (59 strains, 27 characteristics) (Fig. 1). The resultant dendrogram permitted the ad hoc definition of 6 groups (A to F) (cut-off ~ 60%). Clinical and wastewater isolates clustered together, and 6 isolates were unclustered (Fig. 1 and Table S3). These results suggest that wastewater isolates, regardless the type of wastewater, retained clinically relevant features.

Aiming to better explore these results, 22 isolates included in clusters A, B, D, E and F (Fig. 1) were selected for whole genome sequence analysis (Table S4). These were 7 of wastewater (Portugal, 4 RWW, 2 tTWW and 1 hospital effluent), 11 of patients (7 Portugal; 4 Spain) and 4 of clinical environment (Spain). This selection comprised isolates with clinically relevant traits, such as trans-species conjugation and MDR transfer, meropenem resistance, biofilm formation capacity and distinct *G. mellonella* infection indices (0–9). Group C, composed of three isolates unable to conjugate with *E. coli* J53 and not yielding any of the plasmid replicon types tested, were not included in this analysis, mainly interested in assessing clinically-relevant features occurring in distinct lineages.

### Comparative genome analyses

**Fig. 2:**
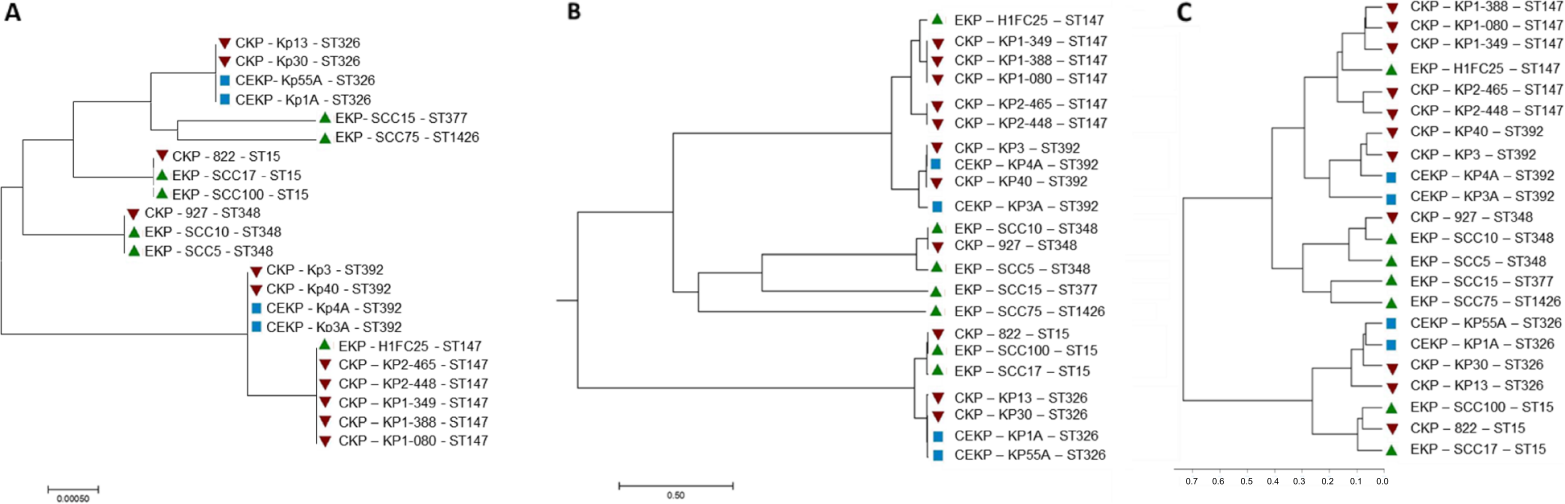
Genome-based phylogenetic analysis of the 22 3^rd^ generation cephalosporin-resistant *K. pneumoniae* isolates. **A** Maximum Likelihood Tree based on the concatenated MLST gene sequences (*gapA, infB, mdh, pgi, phoE, rpoB, and tonB*); **B** UPGMA dendrogram based on ANIb pairwise values comparisons among the genomes, and **C** UPGMA dendrogram representing the degree of similarity of the genomes based on the amino acid sequences presence or absence. Red, green and blue symbols in the dendrogram refer to isolates obtained from patients, wastewater and clinical environment, respectively

The 22 isolates were affiliated to 7 multi-locus sequence types (MLST), 3 of which included wastewater and clinical isolates (ST348, ST15, ST147). Two were represented by single wastewater isolates (ST377 and ST1426). The other two corresponded to the Spanish outbreak isolates (ST326 and ST392), each with two clinical and two environmental isolates (Fig. 2A).

The dendrogram produced based on the average nucleotide identity values (98–100%) (blast algorithm, ANIb) supported the affiliation to 7 MLST groups (Fig. 2B). Also, the comparison of the genomes based on the presence/absence of annotated deduced amino acid sequences supported the formation of the same groups (Fig. 2C). The three types of analyses evidenced the closest relationship between ST15 and ST326, and ST147 and ST392. This organization did not coincide with that reported in Fig. 1, with isolates of the ST147 and ST348 divided by different groups (D and E; and A, E and F, respectively), while others were clustered in groups A or B (ST326, ST392 and ST15, respectively).

**Fig. 3:**
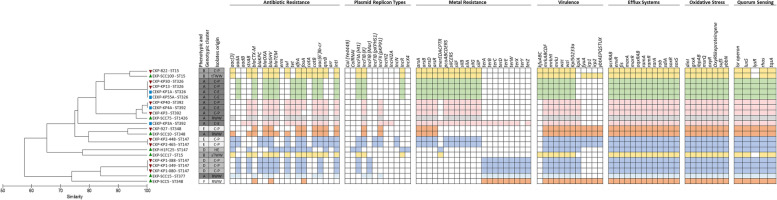
Clustering analysis of 22 3^rd^ generation cephalosporin-resistant *K. pneumoniae* based on selected clinically relevant features (genes encoding antibiotic resistance, metal resistance, virulence, efflux systems, oxidative stress, quorum sensing and plasmid replicon types). A presence/absence table supported the Jaccard similarity index estimation and UPGMA clustering analysis. Colors in the table refer to the trait presence and different colors refer to different isolates MLST. Red, green, and blue symbols in the dendrogram refer to isolates obtained from patients, wastewater, and clinical settings, respectively. C-P – clinical isolate obtained from patients; C-E – isolate obtained from clinical environment; HE – hospital effluent; RWW – raw influent wastewater; sTWW – secondary treatment effluent wastewater; tTWW – tertiary treatment effluent wastewater

A further analysis focused on genetic determinants related to efflux systems, oxidative stress, quorum sensing, virulence, plasmid replicon types and antibiotic and metal resistance genes, as these may be associated to opportunistic pathogens or acquired traits. The genetic determinants related to efflux systems, oxidative stress, and quorum sensing detected were common to all the examined genomes (Table S5), irrespective of the origin or genetic lineage (Fig. 3). Also, *mrk* genes, encoding Type 3 fimbriae involved in bacterial adhesion, and *wzi* and *wzc* genes, involved in bacterial capsule production, and the ARG *bla*_SHV_ were observed in the 22 genomes (Fig. 3). However, these were sometimes represented by different variants (Table S5). The presence of genetic determinants encoding antibiotic resistance, plasmid replicon types, metal resistance, and virulence was variable among the 22 genomes. Antibiotic resistance genes rarely observed were *aadB*1 (in ST392, 1 clinical environment), *ermB* (in ST147, 1 hospital effluent), and *arr-3* (in ST147, 2 clinical). In contrast, the genes *bla*_OXA_, *dfrA*, *fosA* and *aac(6′)lb-cr* were common to most of the 22 isolates, with a few exceptions observed in 1 ST348 RWW isolate where *bla*_OXA_, *dfrA* and *aac(6′)lb-cr* were not detected, in 1 ST147 hospital effluent isolate lacking *bla*_OXA_ and *aac(6′)lb-cr*, and in 1 ST392 clinical isolate that did not yield the *fosA* gene (Fig. 3 and Table S5).

The most common plasmid replicon type was IncFIB (K), curiously not detected in 4 genomes of the ST147 (1 hospital effluent and 3 clinical) and in one ST348. The plasmid replicon type IncFIA was observed in more than half of the isolates (*n* = 13), although in any the ST392, ST1426, ST348, or ST377. Two uncommon plasmid replicon types were colRNAI and IncX4, observed in a single hospital effluent ST147 isolate that lacked the IncFIB (K). Two other uncommon replicon types were IncHI2 and IncHI2A detected in the ST392 isolate of the clinical environment in the Spanish outbreak.

Metal resistance genes were associated with tellurium, mercury, arsenic, copper and silver. Tellurium resistance genes, presumably organized in an operon, were observed in the genomes of 6 isolates, 5 of which of Portugal (2 of wastewater, ST377 and ST348 and 3 clinical, ST147). These genes were not present in all isolates of the same ST, suggesting their acquired character. This was confirmed by the occurrence of *ter* genes in a clinical environment isolate of the ST392 recovered during the outbreak. Curiously, this isolate harbored the unique IncHI2 and IncHI2A plasmid replicon types, referred above, although *ter* and these Inc. groups were not in the same contig. The genes putatively constituting the mercury operon were observed in 9 genomes, all (*n* = 4) of the ST326 (clinical and clinical environment), 3 of wastewater (2, ST15 and 1, ST147) and 1 clinical (ST15) and 1 of clinical environment (ST392). It was suggested that *mer* genes were acquired by ST147 and ST392 isolates in clinical context (hospital effluent and clinical environment, respectively). Genes associated with the copper and silver operon were detected in all ST326 and ST392 isolates, being variable among the ST15 (1 clinical and 1 wastewater isolates) and the ST147 (2 clinical isolates). Arsenic-resistance related genes, described as part of the arsenic operon, were detected in all isolates that also presented the copper and silver resistance genes, being a wastewater and clinical ST348 isolates the exception. The other ST348 isolate with origin in wastewater only yielded *ter* genes, and curiously it was the only one that lacked the IncFIB(K) plasmid replicon (Fig. 3). These observations suggest that in some cases the metal resistance genes were acquired, as they varied within the same genetic lineage and were associated with plasmid replicon types. However, these genes were not lost in wastewater, not even after treatment.

Virulence genes related with iron transport, including siderophore production and capsular serotype K2 were variable among the examined genomes, although these variations were mainly associated with phylogenetic lineages. The only exceptions were observed for isolates of ST15 and ST147. The virulence genes *kfu*, related to iron transport system, were detected in all the isolates (*n* = 7) of the ST15 and ST326. The presence of the genes *fyuA, irp* and *ybt* associated with yersiniabactin siderophore production, detected in 6 isolates, was variable among and within lineages, suggesting its acquired character. These genes were observed in all ST348 isolates, in 1 (out of 3) ST15 isolates, and in 2 (out of 6) ST147 isolates. Interestingly, these 2 ST147 isolates were those yielding the arsenic, cooper and silver resistance genes, while lacked mercury or tellurium resistance genes, present in other genomes of the same ST147. The genes *kpiA* were observed in all isolates (*n* = 15) of ST392, ST1426, ST348, ST147, and ST377 (Fig. 3).

### Antibiotic and metal resistance and virulence genes genetic context

**Fig. 4:**
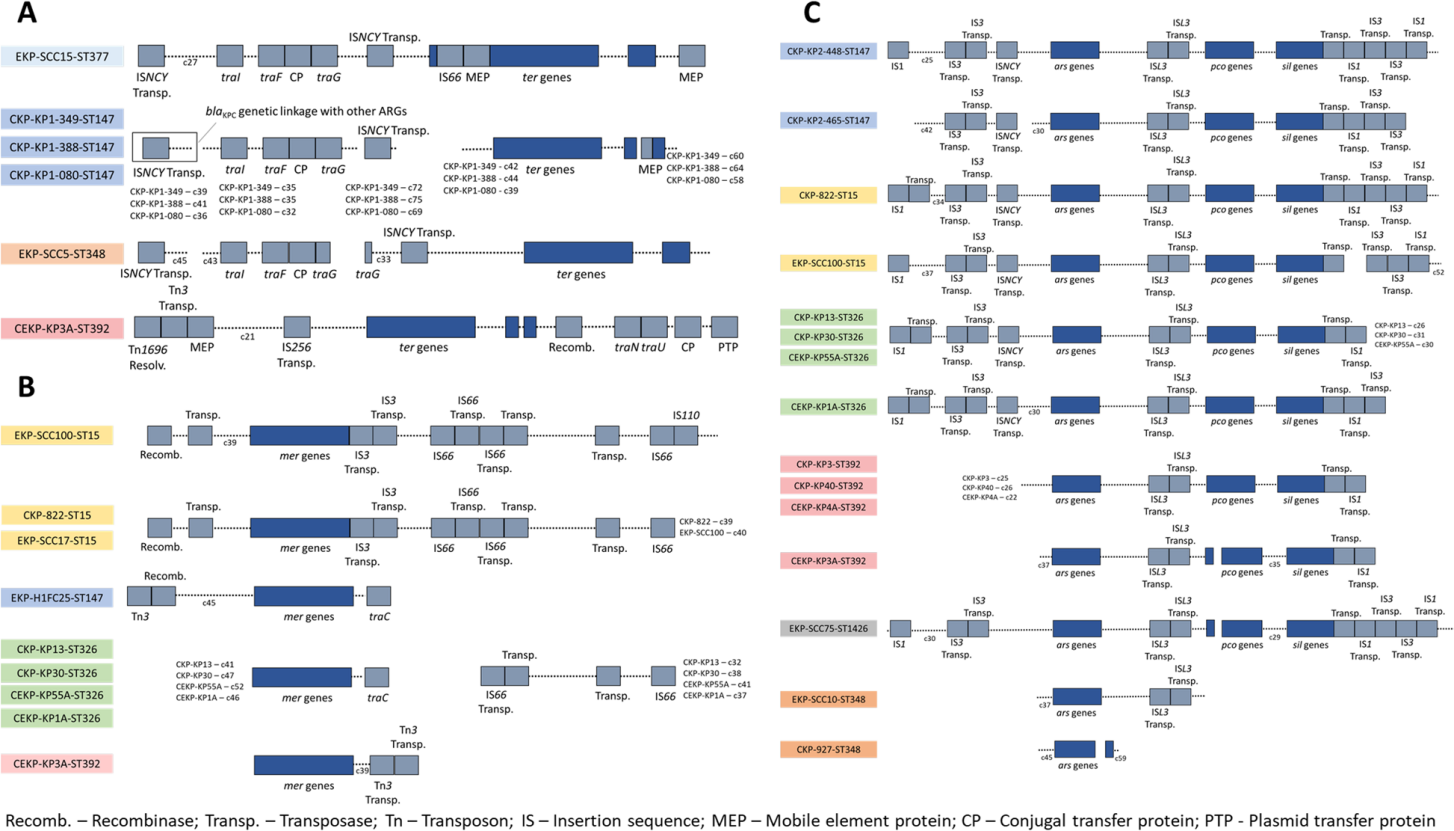
Schematic presentation of the genetic environment of **A** tellurium (*ter*), **B** mercury (*mer*) and **C** arsenic (*ars*), copper (*pco*) and silver (*sil*) resistance-related genes. The contig number (c) is indicated for each isolate close to the schematic presentations

The genetic context of acquired features, whose presence spanned distinct genetic lineages or varied within a single lineage was investigated. These comprised antibiotic (i.e. *bla*_CTX_ e *bla*_KPC_) and metal (*ter*, *mer*, *sil*, *pco*) resistance, and yersiniabactin-related virulence genes. The tellurium-related genes were associated to insertion sequences of the families IS*66* and IS*NCY* in ST377 (1 RWW), of the family IS*NCY* in ST147 (3 clinical) and in ST348 (1 RWW) and of the family IS*256* in ST392 (1 clinical environment) (Fig. 4A, Fig. S1). All the *ter* genes examined presented 99–100% sequence identity with other genomes of *K. pneumoniae* available in public databases, except in the clinical environment isolate (KP3A) recovered during the hospital Spanish outbreak (ST392). In this case, the *ter* genes presented 65–83% nucleotide sequence identity with the others analysed in this study. Moreover, this was the only isolate were the *ter* genes were associated to the Tn*3* and IS*256*. Based on BLASTn search these tellurium operon genes hinted high sequence identity with similar genes observed in the genomes of bacteria of the genera *Citrobacter*, *Enterobacter* and *Salmonella*. In the 6 genomes containing the *ter* genes were also identified *tra* genes, reported as necessary for bacterial conjugation [16]. These *tra* genes were *traI*, *traF*, *traG* in the 5 genomes with the *K. pneumoniae ter*-type, different from the *traN* and *traU* observed in the genome with the atypical *ter* genes (Fig. 4A). Moreover, only in this latter atypical genome, other metal resistance genes (mercury, arsenic, copper, and silver) were observed beside the *ter* genes (Fig. 3, Fig. 4A, B and C).

Mercury-related genes were observed in distinct contexts. In the 3 ST15 isolates and in 1 out of 3 ST392 isolates, those genes were flanked by transposases. In the single ST147 isolate where this gene was detected, it was flanked by a recombinase and a gene involved in conjugation (*traC*), also observed to flank *mer* genes in the 4 ST326 isolates (Fig. 4B). Therefore, depending on the sequence type of the isolates, the mercury-related genes were flanked by different mobilization genes. The genes related to the metals arsenic, copper, and silver were linked in the same contig in most of the isolates (11 out of 13 isolates) and their acquisition through horizontal gene transfer was suggested by their association to transposases in ST147, ST15, ST326, ST392 (Fig. 4C, Fig. S2). In the ST1426 isolate, these genes were not in the same contig (Fig. 4C, Fig. S2). In those 11 genomes, the silver-related genes were flanked by a transposase and the copper-related genes by an IS*L3* transposase. The arsenic-related genes were flanked either by both IS*NCY* and IS*L3* transposases (*n* = 8) or only by IS*L3* transposases (*n* = 3). Two out of the 3 ST348 isolates presented arsenic-, but not copper- or silver- related genes, and in 1 out of 2 isolates these genes were associated in the same contig in an insertion sequence (Fig. 4C, Fig. S2).

**Fig. 5:**
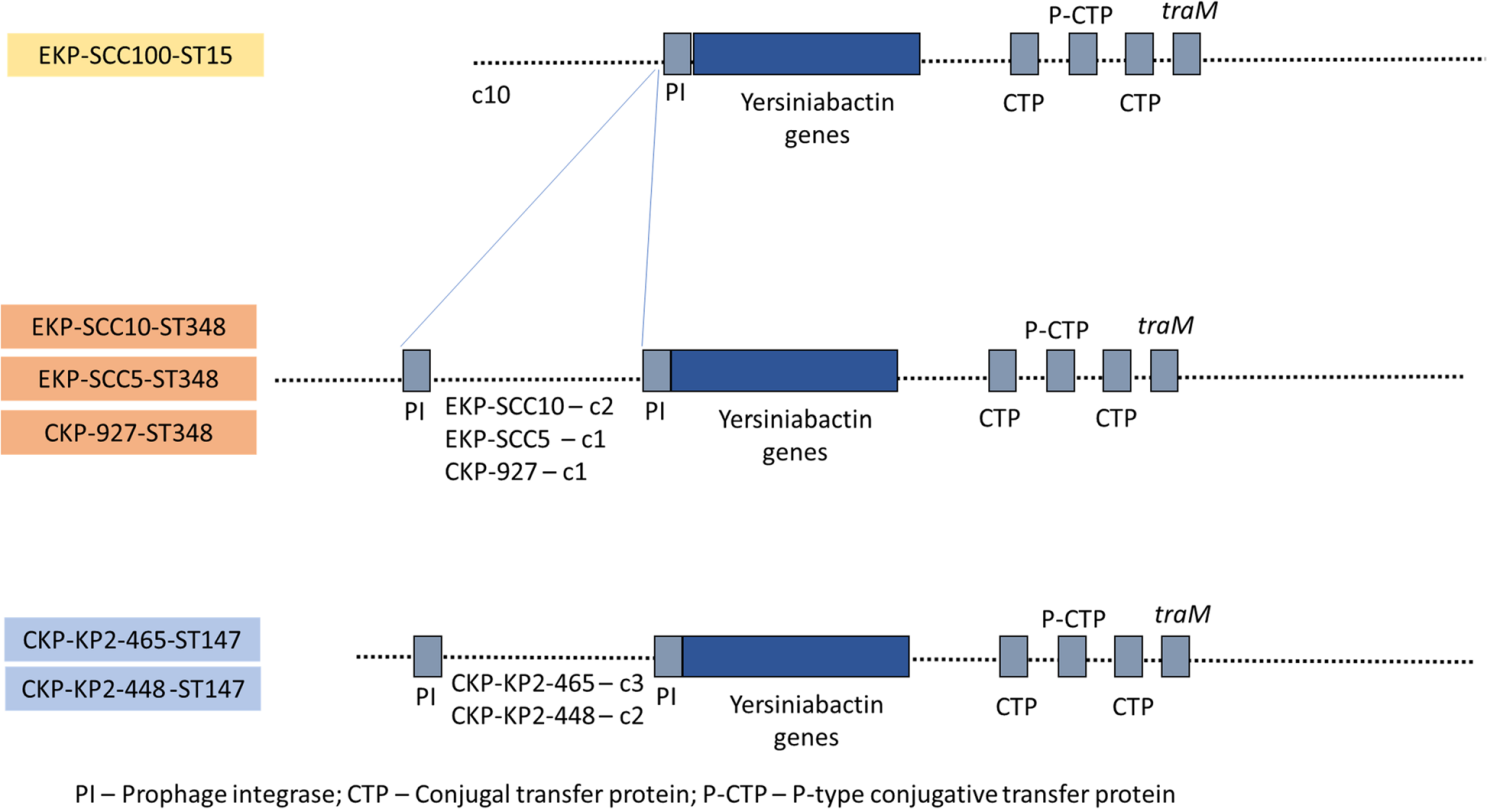
Schematic presentation of yersiniabactin virulence factor genetic environment. The contig number (c) is indicated for each isolate in the cases where more than one isolate shares the same yersiniabactin genetic environment (e.g EKP-SCC10 – c2)

The yersiniabactin *locus*, involved in siderophore production (*fyuA, irp1, irp2, ybtA, ybtE, ybtP, ybtQ, ybtS, ybtT, ybtU* and *ybtX*), was observed to be linked to a prophage integrase and to P-type conjugative transfer protein and *traM* in ST15, ST147 and ST348 (Fig. 5, Fig. S4). The yersiniabactin siderophore production related genes were inserted in integrative and conjugative elements (ICE), whose organization varied according to the sequence type of the isolates. These genes were inserted in ICEkp4 (99.97% of identity with ICEkp4) in the ST15 isolate, in ICEkp5 (99.49% of identity with ICEkp5) in the ST348 isolates, and in ICEkp12 (99.99% of identity with ICEkp12), in the ST147 isolates.

**Fig. 6:**
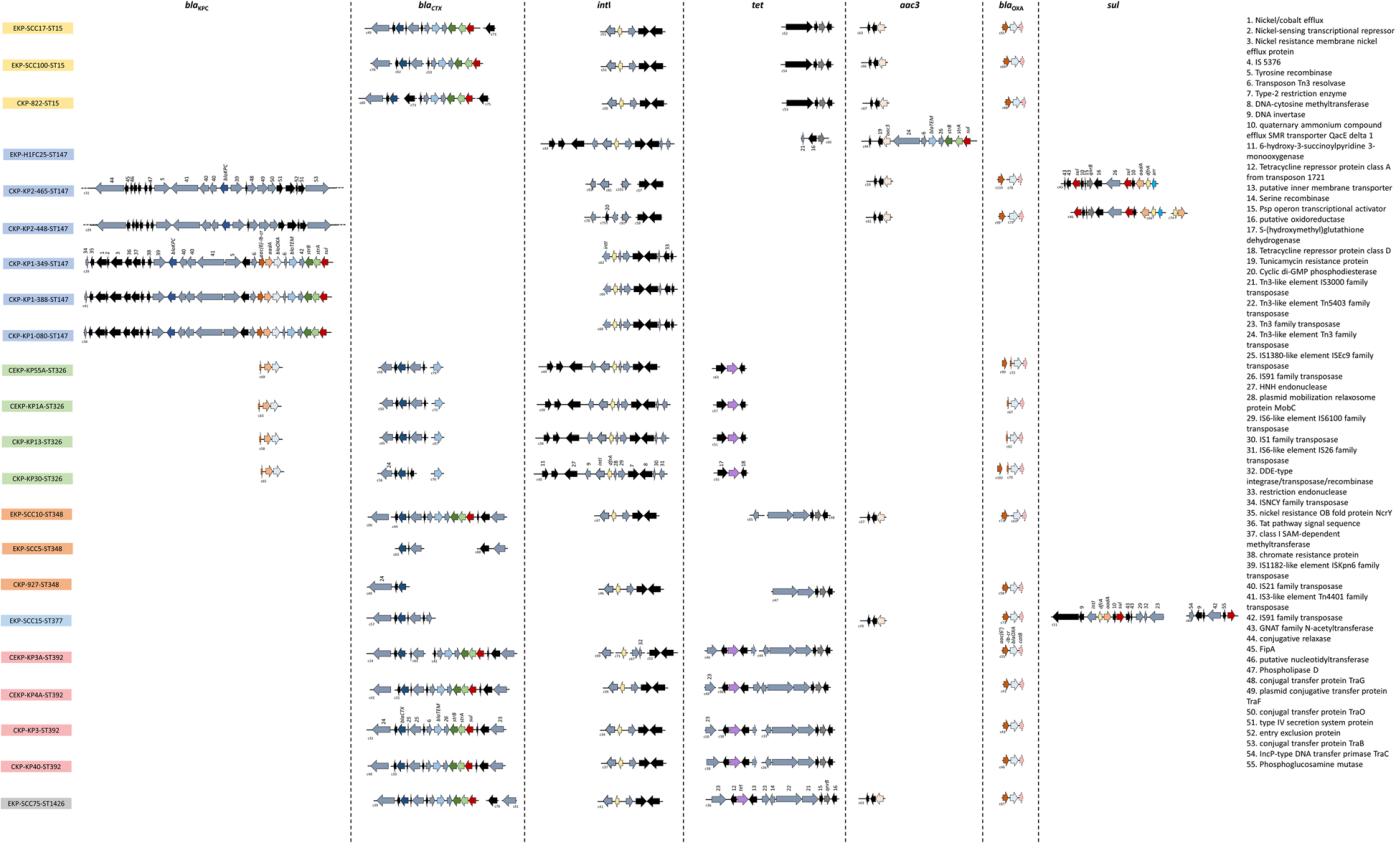
Genetic environment of the genes encoding resistance to carpanemens (*bla*_KPC_), cephalosporins (*bla*_CTX_), tetracyclines (*tet*), aminoglycosides (*aac3*), β-lactams (*bla*_TEM_), and sulfonamides (*sul*). The genetic environment of class I integron (*int*) encoding genes is also presented. The contig number (c) is indicated for each isolate next to the contig that is represented

In general, as expected, the most variable genome features investigated within each phylogenetic group were the plasmid replicon types and the antibiotic resistance genes profile (Fig. 3). The genetic linkage of antibiotic resistance genes was investigated aiming to find hints of distinct acquisition paths. The *bla*_CTX-M-15_ gene, encoding antibiotic resistance to cephalosporins, was observed to be flanked by insertion sequences and/or transposases in all the genomes where this gene was detected (16/22). The *bla*_CTX-M-15_ gene was associated to genes encoding resistance to β-lactams (*bla*_TEM_), aminoglycosides (*strB*, *strA*), and sulfonamides (*sul*) in ST392, ST15, ST348 and, ST1426 (Fig. 6). The gene *bla*_KPC_, encoding resistance to carbapenems was observed to be flanked by transposases in the clinical isolates of the ST147 (*n* = 5). The *bla*_KPC-3_ gene in 2/5 clinical isolates was also associated in the same contig with genes involved in conjugation (Fig. 6), as has been reported before [17]. In the other 3/5 clinical isolates the *bla*_KPC-3_ gene was associated with genes encoding resistance to quinolones (*aac(6′)-lb-cr*), aminoglycosides (*strA* and *strB*), β-lactams (*bla*_OXA-1_/ *bla*_OXA-9_, *bla*_TEM-1A_) and sulfonamides (*sul1*/ *sul2*) (Fig. 6 and Table S5).

## Discussion

As pathogens causing an infection or thriving in the environment, bacteria can be exposed to distinct conditions, which hypothetically may be associated with the retention or loss of specific traits either phenotypic or genotypic [18]. This hypothesis was behind the comparative characterization made in this study. The phenotypic and genotypic characterization organized the isolates according to the antibiotic resistance traits, being resistance to tetracycline, meropenem, sulfonamides and amoxicillin with clavulanic acid the most differentiating. Other differentiating traits were the capacity to conjugate with *E. coli* J53 and transfer antibiotic resistance genes and the biofilm forming capacity. All the features that differentiated the groups have been observed in clinical and in environmental strains [19, 20]. The comparative analysis revealed that the origin of the isolates was not determinant for group organization, although most of the clinical isolates clustered in group A (Fig. 1). This group (A) was characterized by moderate/strong biofilm forming capacity, presence of β-lactamase encoding genes (*bla*_CTX_, *bla*_OXA_, *bla*_SHV_ and *bla*_TEM_), resistance to tetracycline, presence of a single plasmid of high molecular weight and of the replicon type FIIK, as well as the capacity to transfer ARGs and MDR to *E. coli* J53. Also, characteristics such as the capacity of the isolates to conjugate with *E. coli* J53 or to form biofilm were significantly more frequent in clinical or in wastewater isolates, respectively. These observations may suggest a certain degree of habitat adaptation. For instance, the trans-species conjugative capacity described in *K. pneumoniae* [21, 22], may be favored in habitats shared by different species, as is the case of *E. coli* in the gut of patients under antibiotherapy [1, 23]. In turn, the capacity to form biofilm may represent an advantage for nutrient capture or stress protection favoring wastewater bacteria with those properties [3]. Trans-species conjugation and biofilm formation were apparently widespread over the distinct genetic lineages, although with curious exceptions - ST15 strains were not observed to conjugate with *E. coli* J53 and some clinical ST147 strains did not form biofilm. However, these differences are probably not associated with the origin of those strains, whose ubiquity is suggested in the literature, with the ST15, ST147 and ST348 described in clinical and environmental sources [6, 24–28]. The ST326, ST392, ST377 and ST1426 have been reported in other studies as being associated to the clinical settings [29–31]. Indeed, ST326 and ST392 were the lineages recovered from an outbreak in a hospital, and the capacity of a clinical environment ST392 isolate to acquire metal resistance genes was strongly suggested in this study. Also, to our knowledge, ST377 and ST1426 were never reported in wastewater.

Genes related to efflux, oxidative stress or quorum sensing were detected in all the 22 isolates examined. This observation agrees with the literature that identify some genes related with efflux and quorum sensing as part of the *K. pneumoniae* core genome [5, 32]. An exception was the regulator *lysR* gene not detected in isolates of the ST15, which is involved in quorum sensing, oxidative stress response, and has also been associated to the regulation of virulence factors, mainly to the expression of adhesins in early stages of the biofilm formation, being important in the process of infection [33, 34]. This observation is in agreement with the ongoing discussion about the truncated nature of this regulator in members of this lineage [35, 36]. Interestingly, these isolates were moderate/strong biofilm producers and were not able to infect *G. mellonella*, suggesting the importance of *lysR* for infection. The genes *mrk*, *wzc*, *wzi* and *bla*_SHV_ were also observed in the 22 genomes analysed, which is in agreement with previous observations due to its localization in the chromosome [3, 5, 37].

Genes whose detection varied among the examined genomes were related with antibiotic and metal resistance or virulence. Antibiotic resistance genes *bla*_KPC_ and *bla*_CTX_ were genetically linked to other ARGs and were observed to be mainly associated with transposons of the types Tn4401 and Tn3-like. According to the literature, these transposons are widespread in *K. pneumoniae* and are often related with acquisition of *bla*_KPC_ and *bla*_CTX_ [38, 39]. Metal resistance-related genes were associated with the insertion sequences IS66, ISNCY, ISL3, IS3 or IS1, in a pattern shared by isolates of different lineages or origins, except one isolate of the ST392 in which was detected the IS256. The fact that these metal-related genes were flanked by insertion sequences suggests the potential for mobilization [40, 41]. One ST392 isolate was the only Spanish outbreak strain where the *ter* genes were detected, and it presented a unique sequence and context when compared to the other isolates included in this study. Since this was an outbreak isolate it may suggest different paths of gene acquisition from other species, due to selective pressures, as is hinted by the high sequence similarity with homologous genetic elements reported in other genera.

Among the virulence genes detected, genes related to the production of yersiniabactin siderophores, which enable iron acquisition from the host to survive and propagate during the infection process [3], were observed in some ST15 and ST147 and in all ST348 isolates. The yersiniabactin *loci* were associated with integrative and conjugative elements (ICE), as described by Lam and colleagues [42]. Among these, ICEkp4, ICEkp5 and ICEkp12 were specific of the sequence type of the isolates, considering that the first was observed in ST15, the second in ST348 and the third in ST147. This is in agreement with the literature, although in the same sequence types other ICE associated with virulence genes have been reported [42]. Nevertheless, the results suggested that the phylogenetic lineage, more than the origin of the isolates, might explain the paths of acquisition of virulence genes. ICEkp are typically constituted by a P4-like integrase gene in the left end, followed by the yersiniabactin locus, and by a mobilization module constituted by the *xis* excisionase, *virB*-type 4 secretion system (T4SS), *ori*T transfer origin and *mobBC* proteins (responsible for mobilization) [42]. In some ICE structures it is also found a zinc and manganese metabolism module [42], observed in our study in ICEkp4, ST15 isolates.

In this study it was observed that genes related to antibiotic or metals resistance were flanked by insertion sequences, transposases or genes involved in bacterial conjugation. Moreover, genes related to virulence were flanked by ICEs. This observation suggests that the mobilization of the three types of genes uses different mechanisms. Moreover, the genetic analysis of the mobilization structures suggests that the genetic lineage, rather than the source of isolation, are determinant for the genotype and phenotype of the strain. Evidences that isolates of the ST392, ST147, ST15 and ST348 have the potential to acquire or lose metal resistance genes and that such a capacity is related with some plasmid replicon types was evidenced in this study despite the limited number of genomes examined.

Plasmids are present in almost all *K. pneumoniae* isolates with a broad range of replicon types associated [6]. Among them, *bla*_CTX-M-15_ is commonly associated to IncFII plasmids that simultaneously carry other antibiotic resistance genes [26]. Indeed, the isolates of the ST377, ST392, ST348 and ST147 that harbored the gene *bla*_CTX-M_, all presented the *bla*_CTX-M-15_ variant and tested positive for the replicon type IncFII. Also, the *bla*_KPC-3_ observed in clinical isolates of the ST147 was previously described as being associated to the replicon type IncN [17], although this and other replicon types are reported as vectors for these carbapenemase encoding genes (IncX3, IncR, IncHI1 and IncI2) [6].

## Conclusions

A major question of this study was whether the isolation habitat, clinical or wastewater, could influence *K. pneumoniae* isolates features. The phenotypic and genomic studies did not evidence features that highlighted specialization to the isolation habitat. The results suggest that clinical isolates once in wastewater may retain clinically relevant traits, even those that were acquired through horizontal gene transfer and were associated with transposons, insertion sequences or integrative and conjugative elements. Moreover, it is suggested that phylogeny, more than the isolates origin, may explain the profile of acquired traits, although genetic variation may occur within the same genetic lineage.

## Methods

### Study structure and bacterial strains

Fifty-nine *K. pneumoniae* isolates, identified based on the 16S rRNA gene sequence and exhibiting resistance to 3^rd^ generation cephalosporins (cefotaxime and ceftazidime) were selected for this study. The selection of these isolates for this study was due to the clinically relevance of *K. pneumoniae* isolates resistant to 3^rd^ generation cephalosporins and due to their ubiquity across clinical and environmental niches. The bacterial isolates were from wastewater (*n* = 25): 3 from hospital effluent, 12 from raw wastewater and 10 from treated wastewater and clinical (*n* = 34): 30 from patients (26 from Portugal, 4 from Spain) and 4 from the respective clinical environment (Spain) (Table S1). Wastewater isolates were recovered in independent events in the Northern region of Portugal between 2011 and 2016 and from a laboratory collection of 49 isolates were selected those that were resistant to 3^rd^ generation cephalosporins, a feature common to all clinical isolates. The clinical isolates were obtained from urine, faeces or blood samples, among others, of hospitalized patients, collected over a period of 18 months, from 2014 to 2016 in Porto, Portugal. Clinical samples, including urine, faeces, blood and haemoculture samples were processed at the hospital in accordance with the manual for good laboratory practices implemented in this health unit. There was no treatment of personal data and the biological samples are not related to any data that allows the identification of individuals. The 8 clinical isolates recovered in Spain were collected during a hospital outbreak in the Balearic Islands from patients (*n* = 4) and from drains and surface (*n* = 4). As it was hypothesized that during infection the isolates are exposed to specific conditions, which are different from the natural environment, the inclusion of the Spanish outbreak isolates was considered interesting to assess whether genome variation could present a distinct pattern when compared to Portuguese clinical isolates. The isolates were classified in 3 categories: clinical when isolated from patients, clinical environment when isolated from hospital settings, and wastewater (Table S1). The choice for wastewater isolates was based on the fact that this comprises a highly competitive and stressful environment, where pathogens are supposed to be eliminated. The phenotypic characterization of the isolates included clinically-relevant traits such as antibiotic resistance phenotype analyses, trans-species conjugation assays, biofilm formation and infection capacity. Genotypic characterization involved the detection of antibiotic resistance genes and the determination of the number and size of plasmids.

### Antibiotic resistance phenotype and genotype

Resistance phenotypes were determined based on the disk diffusion method, incubated for 24 h at 37 °C, as recommended by the Clinical Laboratory Standards Institute [43] for antibiotics belonging to 5 different classes: β-lactams (AMC, amoxicillin with clavulanic acid, 20/10 μg; AML, amoxicillin, 25 μg; ATM, aztreonam, 30 μg; MEM, meropenem, 10 μg); aminoglycosides (CN, gentamicin, 10 μg; AK, amikacin, 30 μg); quinolones (CIP, ciprofloxacin, 5 μg); sulfonamides (RL, sulfamethoxazole, 25 μg); and tetracyclines (TE, tetracycline, 30 μg), the combination sulfamethoxazole/trimethoprim (SXT, sulfamethoxazole/trimethoprim, 1.25/23.75 μg) and trimethoprim (W, trimethoprim, 5 μg) was also tested. Bacterial isolates were classified as resistant or susceptible according to the inhibition zone diameters recommended by CLSI, 2016 guidelines for *Enterobacteriaceae* [43]. The reference strain *Escherichia coli* ATCC® 25922 was included in each assay as quality control. The genes *bla*_CTX_, *bla*_IMP_, *bla*_KPC_, *bla*_OXA_, *bla*_SHV_, *bla*_TEM_, *bla*_VIM_, and *mcr* were screened by PCR using the primers, annealing temperatures and the conditions described in the literature indicated in Table S6. Positive and negative controls were included in each reaction and amplicons were randomly confirmed based on DNA sequence analysis.

### Plasmid analyses

The plasmid replicon types were screened in total DNA extracted from each isolate using the PCR conditions recommended by Carattoli and colleagues [44]. Positive and negative controls were included in each reaction and amplicon sequencing for authenticity confirmation. In addition, the number and size of plasmids were determined by pulse field gel electrophoresis (PFGE), as described by Ferreira and colleagues [45]. Briefly, cell suspensions were prepared in a cell suspension buffer (100 mM Tris-HCl, pH 8; 100 mM EDTA, pH 8) reaching a final turbidity of 1.4–1.5 at 610 nm and cells were lysed in solidified plugs (1% melted SeaKem Gold agarose (Lonza, Switzerland)) using cell lysis buffer (50 mM Tris-HCl, pH 8; 50 mM EDTA, pH 8; 1% N-lauroylsarcosine sodium salt) and proteinase K (20 mg/mL) for 2 h at 55 °C in an incubator with constant agitation (150 rpm). Plugs were washed twice with sterile ultrapure water at 55 °C for 15 min and four times with TE buffer at 55 °C for 15 min with constant agitation and stored at 4 °C until further analyses. DNA in the plugs was digested with S1 nuclease (per plug: 12.5 μL S1 buffer; 50 U S1 nuclease; total volume of 125 μL)(Thermo Scientific, USA) for 30 min at 37 °C. The PFGE was performed using a 1% SeaKem Gold agarose gel run in CHEF III DR System (Bio-Rad, Laboratories, Hercules, CA, United States) with 0.5 X TBE (45 mM Tris-HCl, pH 8.0; 45 mM boric acid; 1 mM EDTA) for 18 h at 14 °C, with an initial switch time of 6.8 s and final switch time of 35.4 s, 6 V, with a 120° angle. Gels were stained with ethidium bromide (1 mg/mL) for 20 min and distained twice with water for 20 min. The number and size of plasmids harbored by each isolate were determined based on comparisons of the profiles obtained with the molecular weight of bands obtained for the control *Salmonella enterica* serovar Braenderup H9812 digested with XbaI (Thermo Scientific, USA) [46].

### Conjugation assays

The trans-species conjugation assays used *Escherichia coli* J53 as recipient strain. *E. coli* J53 was selected as recipient strain as it was considered a better discriminating feature while normalized possible bias due to intraspecies genetic proximity between donor and recipient. Donors and recipient were cultured in Luria-Bertani (LB) broth overnight at 30 °C with agitation. Conjugation was performed using a 1:3 ratio of donor and recipient cells inoculated in LB medium supplemented with ceftazidime (2 mg/L) and incubated for 20 h at 28 °C. Putative transconjugants were selected on LB agar supplemented with sodium azide (100 mg/L) and ceftazidime (2 mg/L), incubated overnight at 37 °C. The transconjugants were characterized based on antibiotic resistance phenotypes and genes as described above.

### Biofilm formation

The capacity to form biofilm was tested on flat bottom 96-well polystyrene microtiter plates (Orange Scientific, Belgium), in LB (10 g tryptone, 5 g yeast extract, 10 g NaCl), modified LB (mLB) (5 g tryptone, 2.5 g yeast extract, 1.0 g NaCl) and mLB supplemented with cefotaxime (2 mg/L) media. A volume of 100 μL of cultures grown at 37 °C during 18 h with an optical density of 0.1–0.3 at 610 nm was inoculated in a microtiter plate and incubated at 37 °C during 24 h. After measuring the cultures turbidity at 595 nm, planktonic cells were removed by washing the wells 3 times with of 1X phosphate-buffered saline solution. The sediment, presumable biofilm, was air dried for 15 min and was fixed with 98% (v/v) methanol during 15 min, to be stained with 0.1% of crystal violet solution for 10 min at room temperature. The excess of stain was removed under low running tap water and the dye bound to the adherent cells was suspended in 100 μL of ethanol 95% (v/v), during 15 min at room temperature. The optical density of this solution was measured at 570 nm. All assays were made in triplicate. Biofilm formation capacity was classified into 4 categories: negative (OD ≤ OD_c_), weak (OD_c_ < OD ≤ 2xOD_c_), moderate (2xOD_c_ < OD ≤ 4xOD_c_) and strong (4xOD_c_ < OD). OD refers to the optical density measured in each well and OD_c_ = average OD value for the negative control + 3 x standard deviation of average OD values of the negative control [47].

### Infection capacity

Infection capacity assays used *Galleria mellonella* as model organism. A group of 47 isolates (23 wastewater, 20 clinical and 4 clinical environment) selected based on the capacity to form biofilm (highest scores), the capacity for trans-species conjugation, and/or the detection of 2 or more plasmids by PFGE, were tested as previously described [48]. Plate Count Agar (PCA) overnight cultures, at 37 °C, were suspended in saline solution (0.85% NaCl) at a density of ~ 1 × 10^5^ CFUs/μL. A volume of 5 μL of that suspension was injected into each *G. mellonella* larva, in 10 larvae replicates that were incubated at 37 °C in the dark for 72 h. Test larvae were starved overnight at 37 °C, in the dark, were approximately 2–3 cm in length and had no signs of darkening. The injection site (the hindmost left proleg) was disinfected with ethanol and the injection was performed using a micrometer adapted to control the injection volume onto a micro-syringe [49]. At least three independent experiments for each isolate were performed, therefore for each isolate were tested 30 larvae. As negative control, 10 larvae were injected with 0.85% NaCl in each experiment. After the incubation period, selected because after 72 h may be observed pupa formation [50], the injected larvae were individually examined for survival, movement, cocoon formation and melanization, according to Tsai and colleagues [51]. The *G. mellonella* Health Index Scoring System was used to assess the larvae health status where a score ≥ 9 represents a healthy larva and a score < 9 represents an infected larva [52]. From 0 to 8 scores, the lowest the score, the stronger the infection capacity of the bacterial isolate and consequently more severe was the effect on larva health.

### Genome analysis

A group of 22 isolates (7 of wastewater, 11 clinical, 4 of clinical environment) were selected for whole genome sequencing based on the phenotypic and genotypic characterization performed (Table S4). Representative genomes from the groups determined (Fig. 1) and presenting clinically relevant traits such as moderate/strong biofilm producers, conjugative, presenting multidrug-resistance, among others characteristics were selected for genomic analysis. The genomes sequences were determined using the paired-end Illumina HiSeq, the quality of the reads obtained was checked with the FastQC v0.11.8 software and the genomes were assembled using SPAdes v3.11.1 software. The resulting contigs with low coverage (< 2%), or with a size below 500 bp were removed and the quality of the genomes was assessed using the CheckM method [53].

The coverage of genomes was determined based on the formula C=NxL/G (C, coverage; N, number of reads; L, average read length; G, genome size). The whole genomes shotgun sequences obtained in this study have been deposited in the DDBJ/ENA/GenBank database. Accession numbers are indicated in Table S4. The version described in this paper are the first version. Genomes average nucleotide identity based on the blast algorithm (ANIb) and the percentage in GC was determined using JSpeciesWS online service (http://jspecies.ribohost.com/jspeciesws/#analyse).

For genome comparison whole genomes were annotated using PROKKA version 1.12 [54] and the amino acid sequences obtained for all the functional categories for each isolate were compared using the criteria of 70% similarity over 50% of coverage alignment using the GET_HOMOLOGUES software [55]. Average identity matrixes were calculated with BLASTp scores among the protein sequences of the genomes and a dendrogram representing the degree of similarity of the genomes based on the amino acid sequences presence or absence was obtained.

The internal fragments of a group of 7 housekeeping genes (*gapA*, *infB*, *mdh*, *pgi*, *phoE*, *rpoB* and *tonB*) from the *K. pneumoniae* Multi Locus Sequence Type (MLST) scheme [56] were extracted from each *K. pneumoniae* genome to determine the MLST using the Institute Pasteur database. Concatenated sequences were aligned using MEGA7 and a phylogenetic tree was constructed using the Maximum Likelihood method with a bootstrap of 1000 replicates. Clinically relevant genome features such as the genes annotated as encoding for antibiotic and metal resistance, virulence, quorum sensing, and oxidative stress related functions and plasmid replicon type were screened on the *K. pneumoniae* whole genomes sequences. A total of 127 clinically relevant genes were downloaded from publicly available databases and used to perform BLASTn searches against a database created in house constituted by the genomes in analyses. Metal resistance (*n* = 35), virulence (*n* = 26) and efflux systems (*n* = 17) related genes sequences were downloaded from the Institute Pasteur database (https://bigsdb.pasteur.fr/cgi-bin/bigsdb/bigsdb.pl?db=pubmlst_klebsiella_seqdef&page=downloadAlleles), antibiotic resistance genes (*n* = 21) from ResFinder database [57] and cross-checked with CARD database (https://card.mcmaster.ca/), plasmids replicon type genes (*n* = 12) were downloaded from PlasmidFinder 2.1 tool from Center for Genomic Epidemiology (https://cge.cbs.dtu.dk/services/PlasmidFinder/). Quorum sensing (*n* = 7) and oxidative stress (*n* = 9) related genes were searched in Uniprot database using the terms “*Klebsiella pneumoniae* quorum sensing” and “*Klebsiella pneumoniae* oxidative stress” and downloaded from NCBI database. Considering that the presence of genes encoding metals resistance and yersiniabactin virulence was variable among closely phylogenetically related strains, the genetic context of those genes was explored seeking to unveil possible hints of genes acquisition. The genetic linkage between antibiotic resistance encoding genes was investigated aiming to assess acquisition patterns.

### Statistical analysis

The phenotypic and genotypic characteristics of the 59 *K. pneumoniae* isolates were compared based on the Jaccard similarity index using the software Primer & Permanova v6 (Primer-e, New Zealand) and expressed as a dendrogram obtained with UPGMA algorithm. The data was organized in a 1/0 (presence1; absence =0) table for the following characteristics: 1) PCR gene screening; 2) antibiotic resistance phenotype; 3) number of plasmids; 4) size of plasmids (< 150 Kbp = 0; ≥150 Kbp = 1); 5) plasmids replicon types; 6) conjugation with *E. coli* J53; 7) ARGs acquired in transconjugants (1 gene = 0; ≥2 genes = 1); 8) multidrug resistance acquired by transconjugants (≤2 antibiotic classes = 0; ≥3 antibiotic classes = 1) and 9) biofilm formation in LB and mLB (negative/weak = 0; moderate/strong = 1). Fisher’s exact test, adequate to compare small samples with low frequency values [58], was used to evaluate statistically significant differences between clinical and environmental isolates phenotypic and genotypic characteristics using a *p*-value ≤0.05.

## Supplementary Information

**Additional file 1 **: **Table S1.** 3^rd^ generation cephalosporin-resistant *K. pneumoniae* isolates characterized based on β-lactam and carbapenem encoding genes, antimicrobial resisitance phenotype, plasmid number, size and replicon types, conjugation properties and biofilm formation capacity. A total of 25 isolates were obtained from wastewater, 30 from patients and 4 from clinical settings. **Table S2.** Antimicrobial resistance phenotype and genotype transferred to transconjugants by the conjugative isolates from the 59 isolates of *K. pneumoniae* resistant to 3^rd^ generation cephalosporins. + indicates detection of the trait, − indicates not detection of the trait, S, I and R indicate susceptible, intermediate and resistant to the antibiotic, and NA indicates not assessed because the trait was not detected in the donor cell. **Table S3.** Clusters obtained based on the pheno- and genotypic characteristics of the 3^rd^ generation cephalosporin-resistant *K. pneumoniae* isolates and dominant characteristics observed in each cluster. **Table S4. **3^rd^ generation cephalosporin-resistant *K. pneumoniae* isolates genomes used for comparative genomic analysis. **Table S5.** Genes searched related to clinically-relevant properties on selected 3^rd^ generation cephalosporin-resistant *K. pneumoniae* genomes. 1 indicates the detection of the gene and 0 indicates the not detection of the gene. For each gene it is indicated the allele that was detected, the number of genomes in which it was detected, the length of the gene and of the query, the number of different nucleotides in the query sequence and the number of gaps. The accession number and the database from which each gene allele was downloaded is also indicated. **Table S6.** Primer sequences and PCR amplification conditions used in this study.

**Additional file 2. Figure S1.** Genetic environment of tellurium (*ter*) resistance-related genes. Blue arrows indicate the genes related to tellurium resistance and yellow arrows refer to hypothetical proteins; **Figure S2.** Genetic environment of arsenic (*ars*), copper (*pco*) and silver (*sil*) resistance-related genes. Blue arrows indicate genes related to resistance to arsenic, copper and arsenic metals, yellow arrows refer to hypothetical proteins; **Figure S3.** Genetic context of mercury (*mer*) resistance-related genes. Blue arrows indicate the genes related to mercury resistance and yellow arrows refer to hypothetical proteins; **Figure S4.** Genetic environment of yersiniabactin virulence locus. Blue arrows indicate genes related to yersiniabactin virulence and yellow arrows refer to hypothetical proteins.

## Data Availability

The datasets generated and/or analysed during the current study are available in the NCBI repository: JAGSXY000000000, JAGSZX000000000, JAGSZY000000000, JAGSZR000000000, JAGSZS000000000, JAGSZZ000000000, JAGTAA000000000, JAGTAB000000000, JAGTAC000000000, JAGTAD000000000, JAGTPB000000000, JAGTOX000000000, JAGTOV000000000, JAGTOS000000000, JAGTOO000000000, JAGTON000000000, JAGTOL000000000, JAGTOK000000000, JAGSZT000000000, JAGSZU000000000, JAGSZV000000000, JAGSZW000000000.
