# Supplementary material for: Third generation cephalosporin-resistant Klebsiella pneumoniae thriving in patients and in wastewater: what do they have in common?
Source: BMC Genomics. 2022 Jan 22;23:72. doi: 10.1186/s12864-021-08279-6 (PMC8783465; doi:10.1186/s12864-021-08279-6)
Supplement: Supplementary file 2 — Additional file 2. Figure S1. Genetic environment of tellurium (ter) resistance-related genes. Blue arrows indicate the genes related to tellurium resistance and yellow arrows refer to hypothetical proteins; Figure S2. Genetic environment of arsenic (ars), copper (pco) and silver (sil) resistance-related genes. Blue arrows indicate genes related to resistance to arsenic, copper and arsenic metals, yellow arrows refer to hypothetical proteins; Figure S3. Genetic context of mercury (mer) resistance-related genes. Blue arrows indicate the genes related to mercury resistance and yellow arrows refer to hypothetical proteins; Figure S4. Genetic environment of yersiniabactin virulence locus. Blue arrows indicate genes related to yersiniabactin virulence and yellow arrows refer to hypothetical proteins. [file 12864_2021_8279_MOESM2_ESM.pptx]

## Slide 1
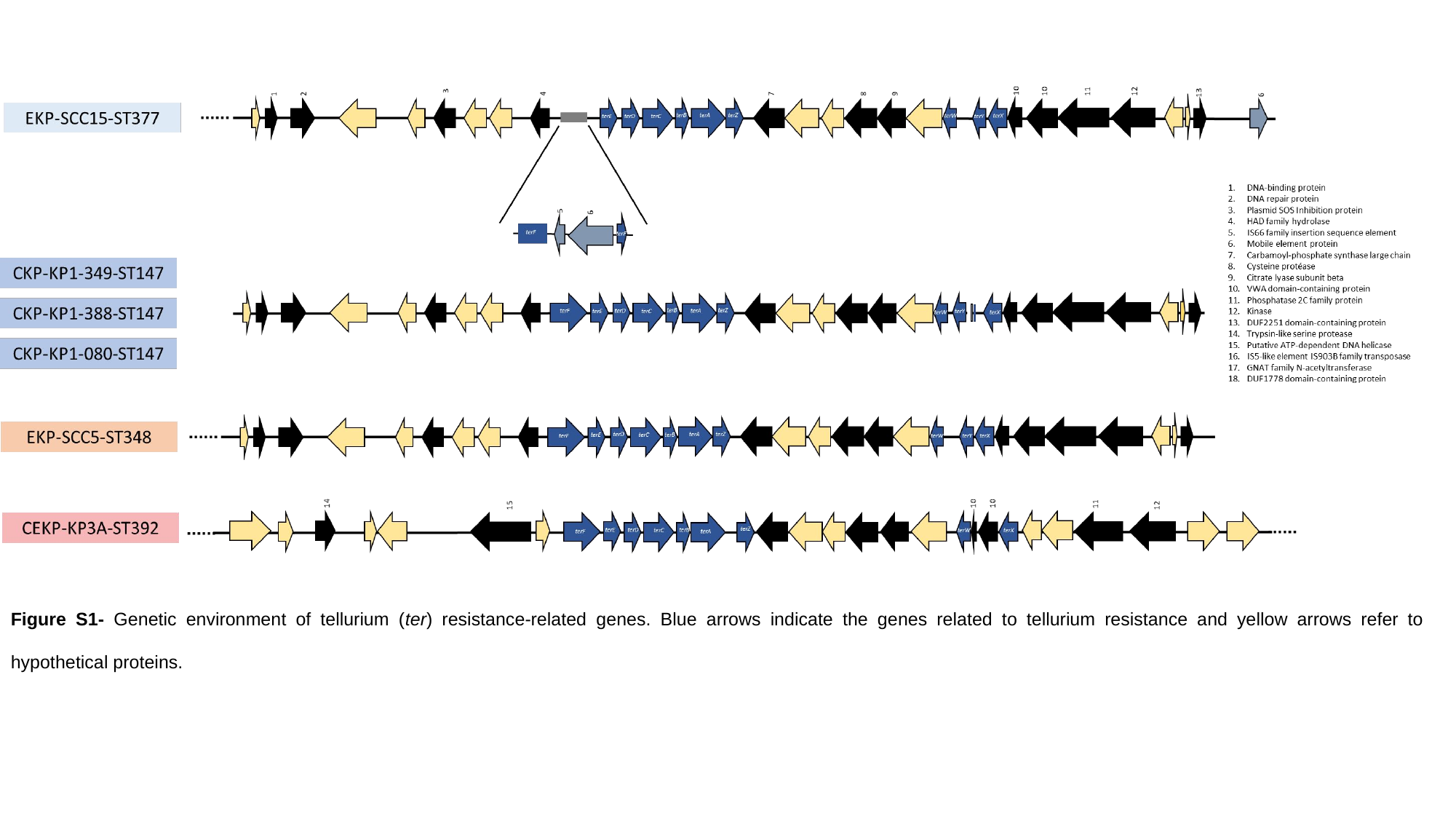

Figure S1- Genetic environment of tellurium (ter) resistance-related genes. Blue arrows indicate the genes related to tellurium resistance and yellow arrows refer to hypothetical proteins.

## Slide 2
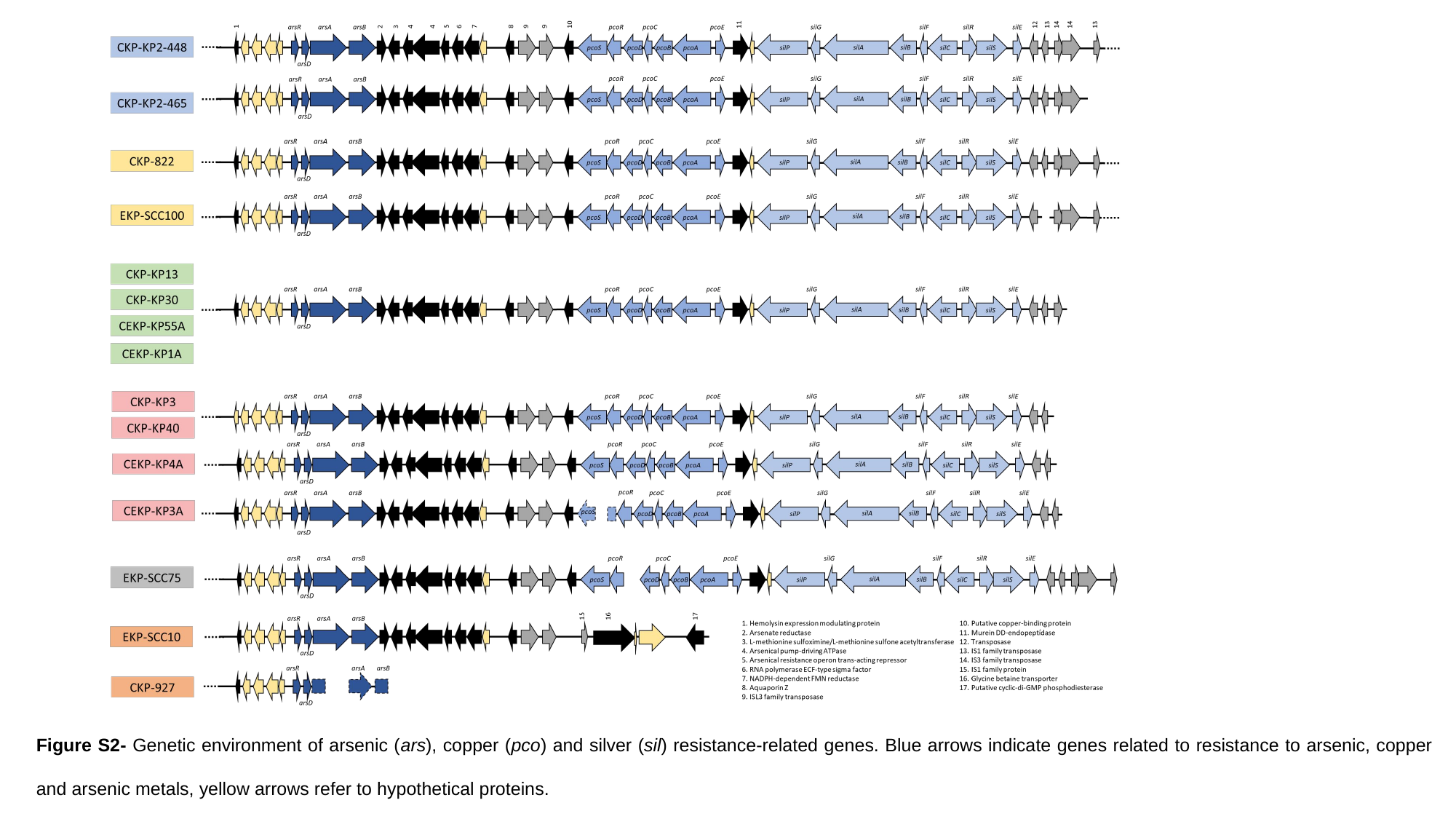

Figure S2- Genetic environment of arsenic (ars), copper (pco) and silver (sil) resistance-related genes. Blue arrows indicate genes related to resistance to arsenic, copper and arsenic metals, yellow arrows refer to hypothetical proteins.

## Slide 3
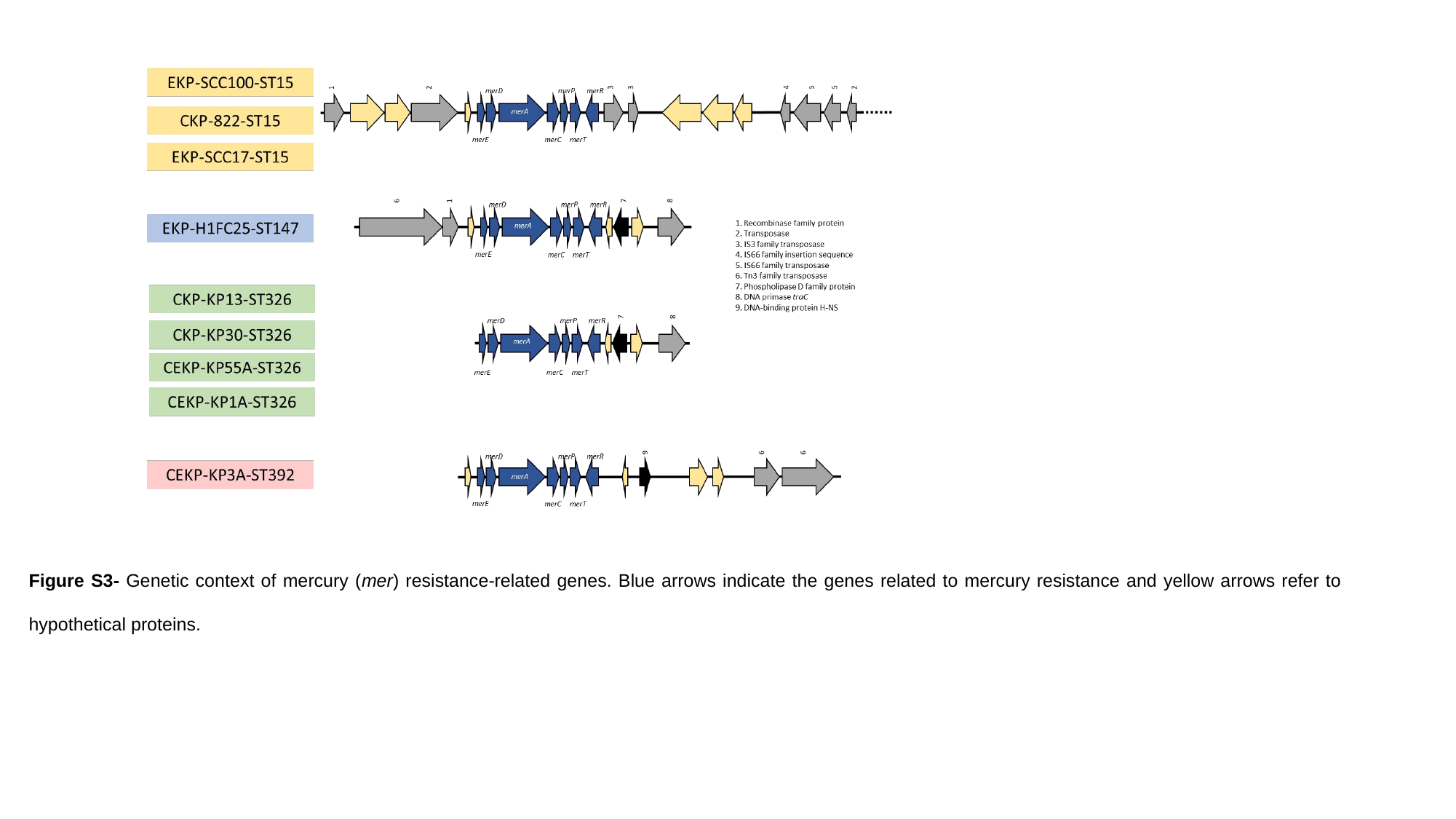

Figure S3- Genetic context of mercury (mer) resistance-related genes. Blue arrows indicate the genes related to mercury resistance and yellow arrows refer to hypothetical proteins.

## Slide 4
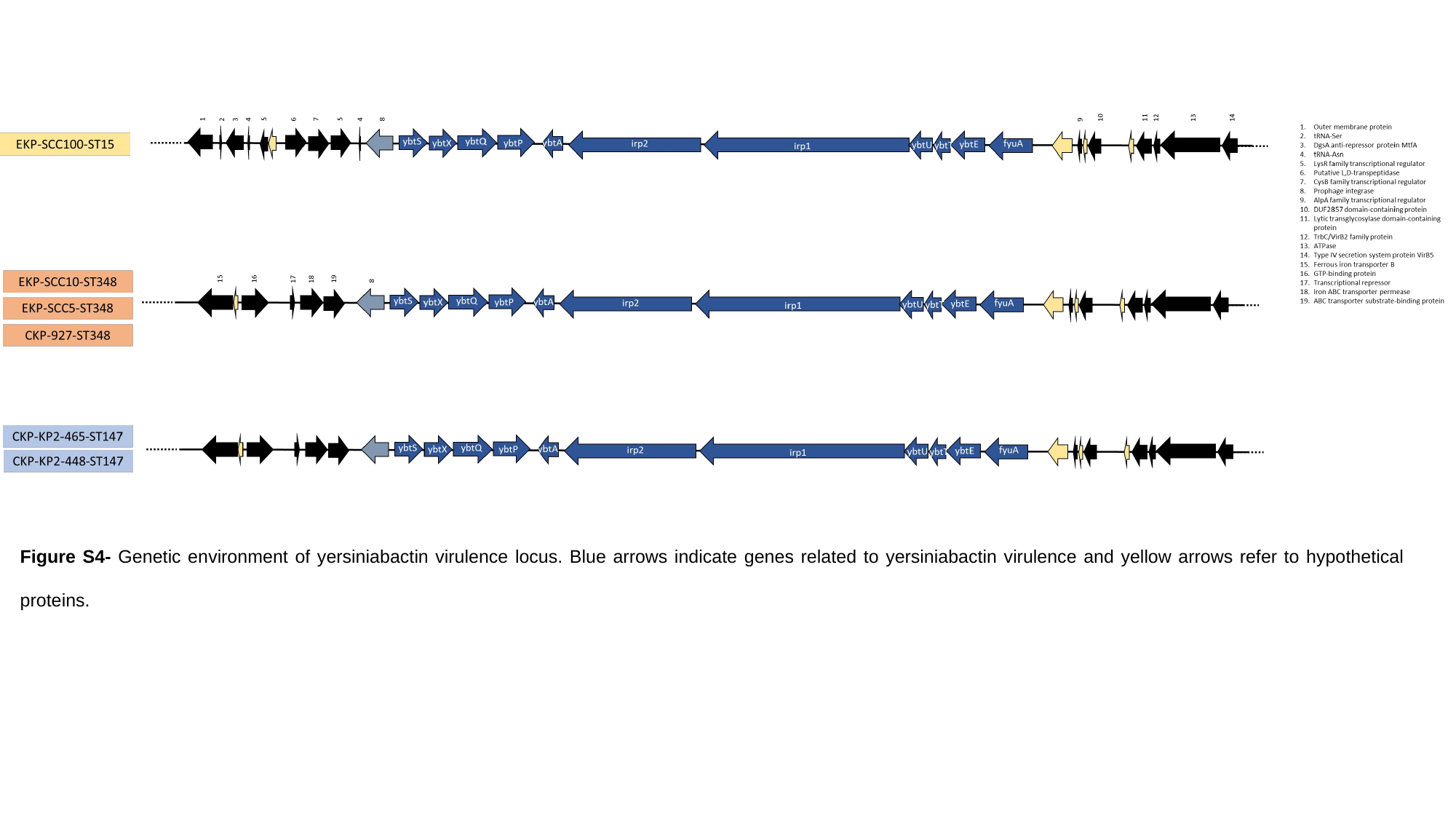

Figure S4- Genetic environment of yersiniabactin virulence locus. Blue arrows indicate genes related to yersiniabactin virulence and yellow arrows refer to hypothetical proteins.
